# Supplementary figures and images for: A Long Noncoding RNA Derived from lncRNA–mRNA Networks Modulates Seed Vigor
Source: Int J Mol Sci. 2022 Aug 22;23(16):9472. doi: 10.3390/ijms23169472 (PMC9409430; doi:10.3390/ijms23169472)

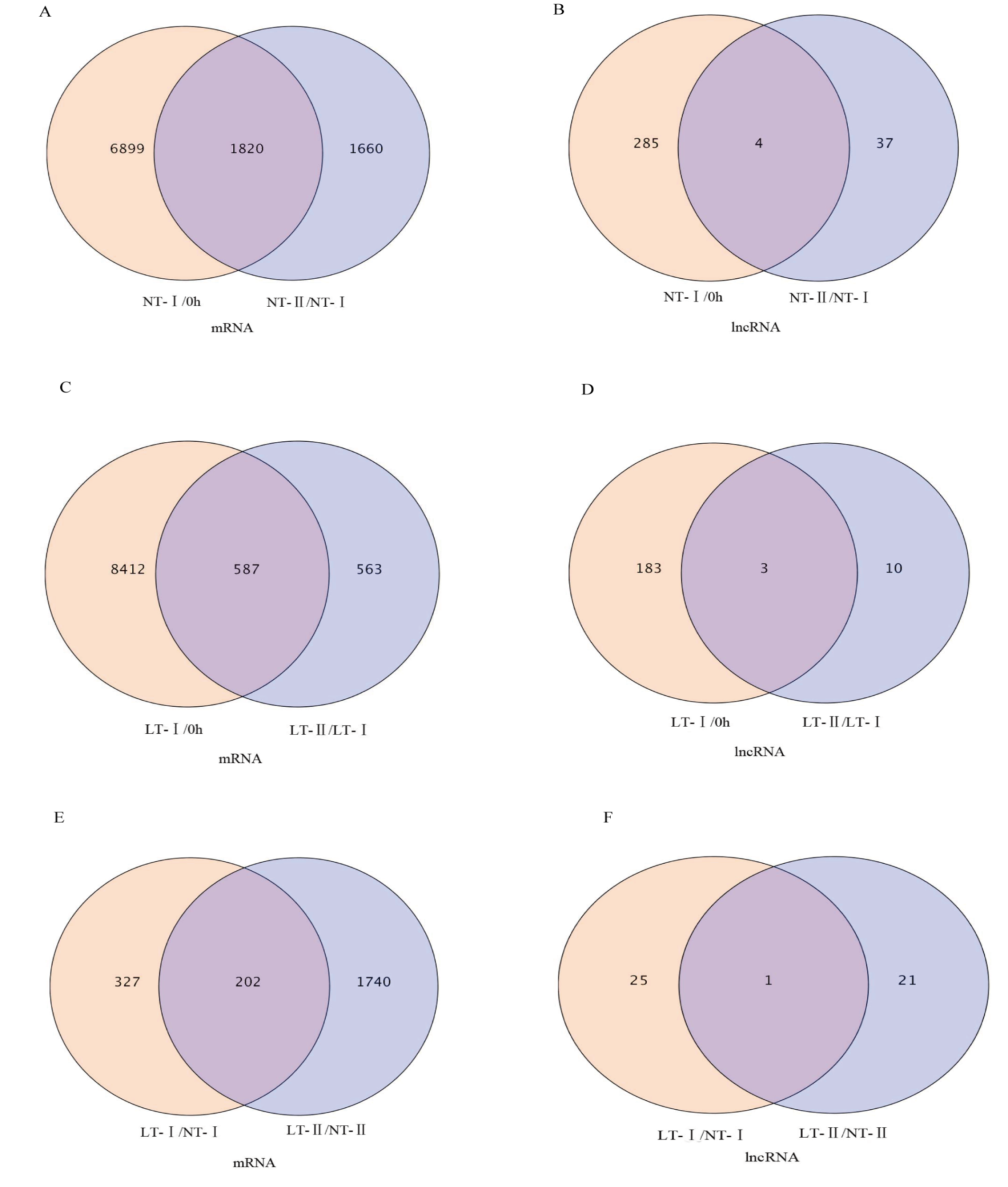

Supplement: Supplementary file 1 [file ijms-23-09472-s001.zip › Supplemental Figure S1.tif]

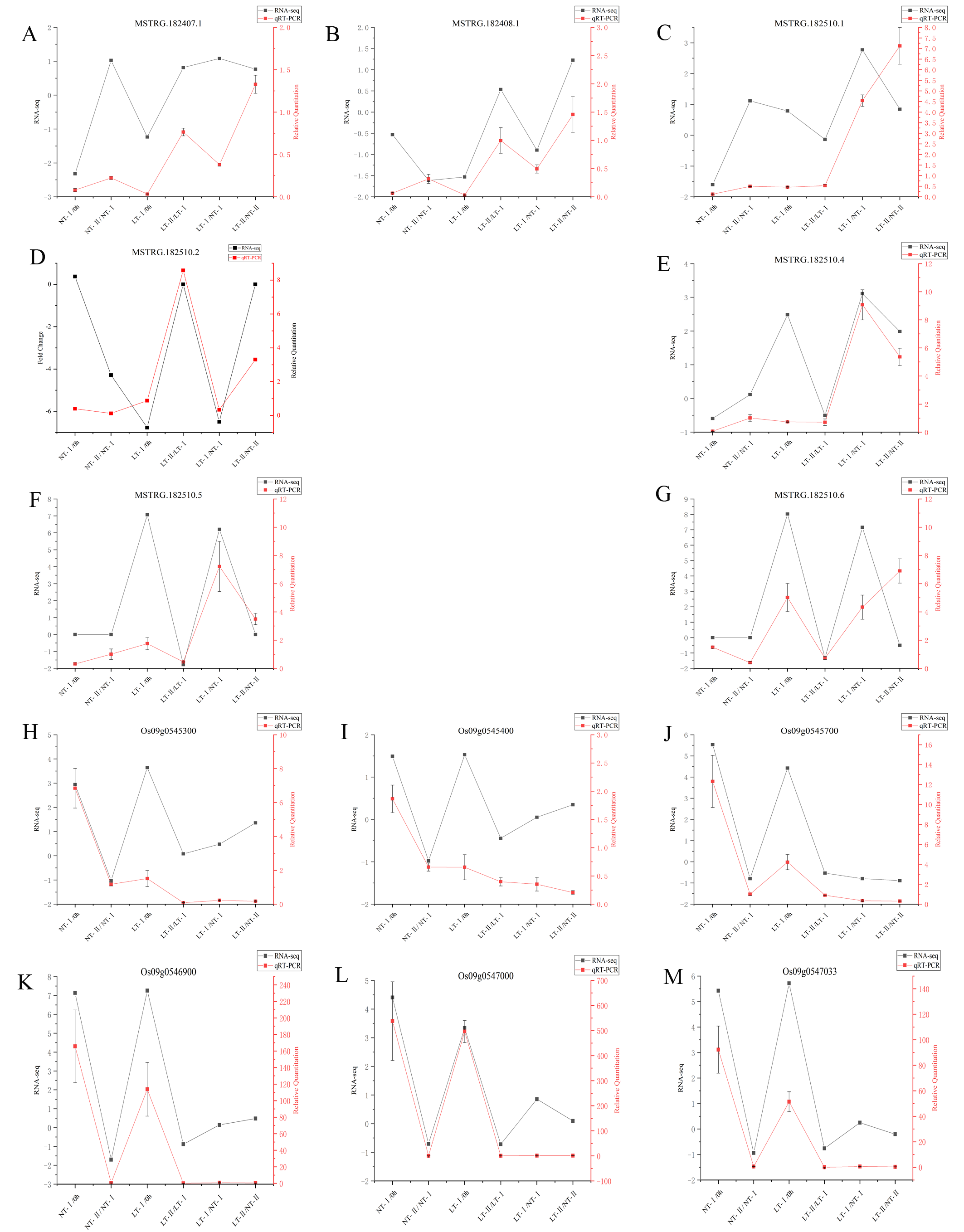

Supplement: Supplementary file 1 [file ijms-23-09472-s001.zip › Supplemental Figure S3.tif]

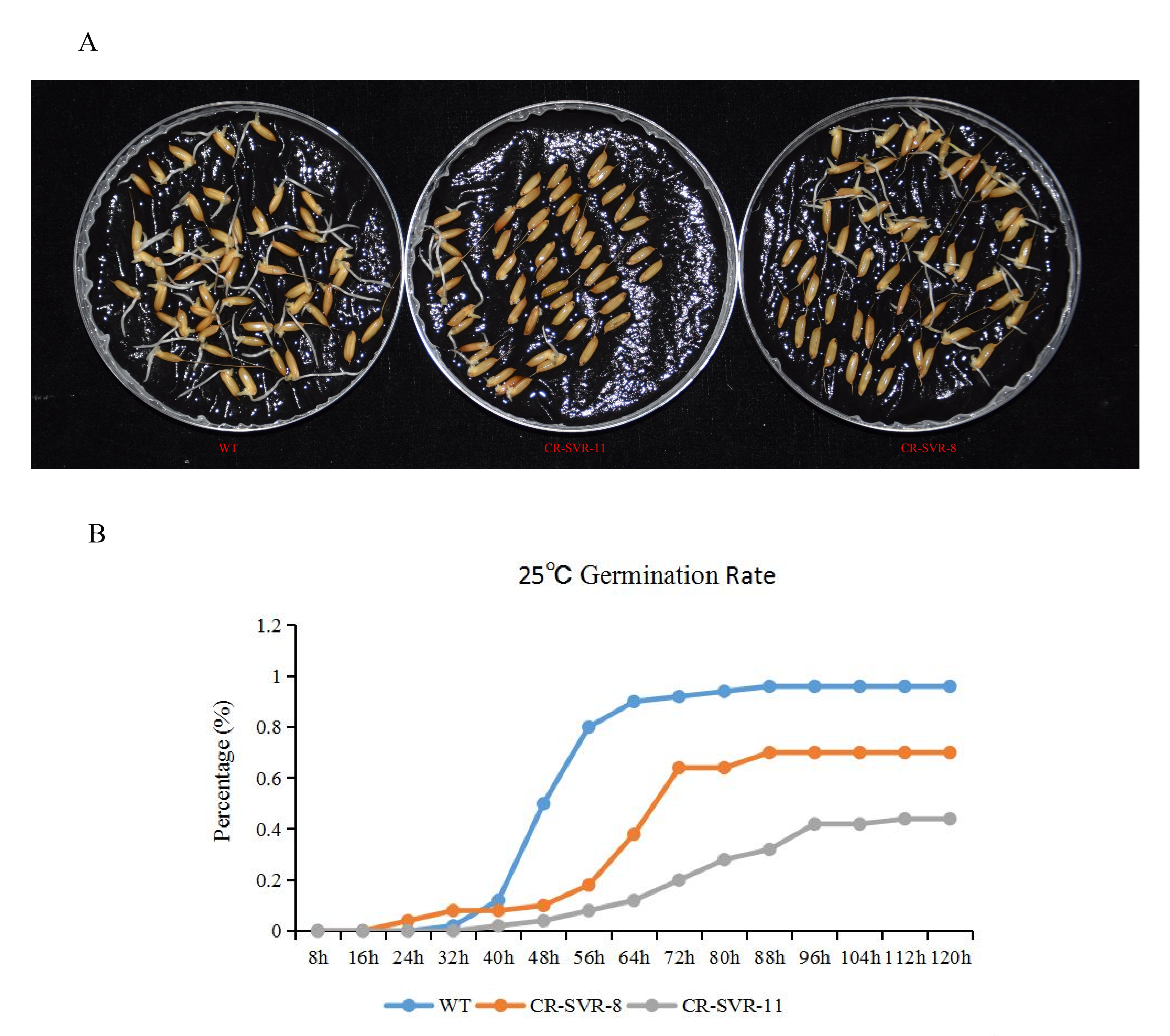

Supplement: Supplementary file 1 [file ijms-23-09472-s001.zip › Supplemental Figure S4.tif]
